# Similarity Among Friends Serves as a Social Prior: The Assumption That “Birds of a Feather Flock Together” Shapes Social Decisions and Relationship Beliefs

**DOI:** 10.1177/01461672221140269

**Published:** 2023-02-02

**Authors:** Miriam E. Schwyck, Meng Du, Yuchen Li, Luke J. Chang, Carolyn Parkinson

**Affiliations:** 1 University of California, Los Angeles, USA; 2 Dartmouth College, Hanover, NH, USA

**Keywords:** social cognition, interpersonal relationships, decision-making, social networks, third-party relationships

## Abstract

Social interactions unfold within networks of relationships. How do beliefs about others’ social ties shape—and how are they shaped by—expectations about how others will behave? Here, participants joined a fictive online game-playing community and interacted with its purported members, who varied in terms of their trustworthiness and apparent relationships with one another. Participants were less trusting of partners with untrustworthy friends, even after they consistently showed themselves to be trustworthy, and were less willing to engage with them in the future. To test whether people not only expect friends to behave similarly but also expect those who behave similarly to be friends, an incidental memory test was given. Participants were exceptionally likely to falsely remember similarly behaving partners as friends. Thus, people expect friendship to predict similar behavior and vice versa. These results suggest that knowledge of social networks and others’ behavioral tendencies reciprocally interact to shape social thought and behavior.

Sayings like “birds of a feather flock together” reflect the widespread intuition that we tend to be surrounded by those who are similar to us. Indeed, similarity between people who are socially close to one another is a common feature of human social networks and can arise from various mechanisms. For example, social influence can result in friends becoming more similar (Altermatt & Pomerantz, 2003; Brechwald & Prinstein, 2011; de Klepper et al., 2010), and people who are similar (e.g., in terms of demographic characteristics) are more likely to become friends due to both structural factors that constrain whom individuals are likely to encounter (i.e., “induced homophily”) and individuals’ preferences (i.e., “choice homophily”; McPherson et al., 2001). Indeed, there is a large body of sociological research examining this phenomenon, particularly focusing on the existence and effects of demographic homophily in real-world social networks (e.g., Lawrence & Shah, 2020; McPherson et al., 2001; Shrum et al., 2016; J. A. Smith et al., 2014). Additional work bridging sociology and psychology has begun to examine how cognitive, affective, and behavioral tendencies are related to friendship, and more generally, proximity in social ties (e.g., Apicella et al., 2012; Dehghani et al., 2016; Ehlert et al., 2020; Fowler & Christakis, 2010; Parkinson et al., 2018; Pradel et al., 2009; K. M. Smith et al., 2018). This body of research complements related work in psychology regarding similarity-based attraction and social influence (e.g., Henderson & Furnham, 1982; Kuwabara et al., 2022; Wetzel & Insko, 1982). Thus, convergent evidence spanning multiple disciplines has linked social closeness to interpersonal similarity with respect to a variety of factors, including how people tend to think, feel, and behave, as well as demographic characteristics.

Given that similarity among friends is commonly observed in human social networks, it may be that people *assume* such similarities exist, and that this assumption serves as a heuristic to inform predictions of how others will behave and to scaffold mental representations of friendships between others. Although people’s perceptions and reality are not always aligned, there have been decades of research on how people cognitively represent their social networks, finding that people often overperceive common characteristics of social networks, including, for example, balanced triads (two people who have a friend in common are often assumed to be friends), and the likelihood that people of the same race are friends compared with individuals of different racial backgrounds (Brands, 2013). Assuming such characteristics would be beneficial, as it would reduce the amount of information that humans need to remember. Moreover, because people cannot exhaustively observe others’ social behaviors and relationships, they may use strategies to fill in the gaps. By characterizing the systematic errors people make in their inferences about others, we can gain insight into such strategies. It is unknown, however, if an assumption of behavioral similarity among friends exists and how such an assumption might shape social thought and behavior. Does knowing how a stranger’s friend behaved affect how we treat that stranger? Furthermore, does this assumption shape our perception of others and our memory of others’ relationships?

Prosocial tendencies provide a useful lens through which to examine these questions for several reasons. Whereas much work on homophily has focused on relatively coarse and often readily observable characteristics (e.g., race and age), some research has argued for homophily in prosocial behavioral tendencies (Apicella et al., 2012), which may be possible to the extent that cooperative tendencies are observable (e.g., learned through one’s own social interactions, observing others’ interactions, or gossip). Indeed, people can accurately learn about others’ tendencies to cooperate or freeload, this information then shapes individuals’ reputations, and people use such reputations to maximize their own gain (Krasnow et al., 2012; Price, 2006). Therefore, it appears that people are keenly attuned to and readily learn about how prosocial others are, and cooperation thus provides a context where people are motivated to do so. In particular, when playing cooperative games in which they themselves have a stake in the outcome, people are motivated to do their best to learn about others’ behaviors and accurately predict their decisions. Thus, cooperative games provide a sensitive lens for examining these mental processes in a controlled context.

Here, we use prosocial tendencies to test whether similarity among friends acts as a social prior (i.e., as a belief people have before taking new evidence into account), leading people to believe that friends are likely to behave similarly (i.e., those who “flock together” are probably “birds of a feather”), and people who behave similarly are more likely to be friends (i.e., “birds of a feather” probably “flock together”). That is, we test two hypotheses about people’s assumption of a similarity–friendship association: (a) assumptions of similarity among friends lead people to believe that friends will behave similarly to one another, and (b) assumptions of similarity among friends lead people to believe that those who behave similarly are particularly likely to be friends.

## Do People Expect Friends to Behave Similarly?

Effective, beneficial interactions with others are critical to individuals’ well-being, as well as to their personal and professional success. However, people also often need to interact with individuals whom they have not previously encountered. Decisions about how to interact with such strangers can lead to impactful consequences. For example, if you ask a stranger to watch your laptop for a few minutes, you may end up mourning the loss of your computer if the stranger proves to be untrustworthy. Many researchers have examined how people infer others’ trustworthiness through direct interactions (Chang et al., 2010; Fareri et al., 2012; Fouragnan et al., 2013), through character knowledge and reputation (e.g., explicit character descriptions, gossip; Delgado et al., 2005; Feinberg et al., 2014; Fouragnan et al., 2013), and by interpreting what physical appearance might signal (e.g., ingroup status, face-based trait attributions; Stanley et al., 2011; Todorov et al., 2009; van’t Wout & Sanfey, 2008; Wilson & Eckel, 2006). Yet, many of the strangers with whom we interact also have relationships with individuals we know, and knowledge of such associations may provide valuable clues regarding how unfamiliar others will behave in the absence of previous interactions or character information. That is, people may make assumptions about how a stranger will behave based on their knowledge of how that person’s friends tend to act. Indeed, there is growing research interest in how we think about and are affected by the social networks that surround us (E. R. Smith & Collins, 2009; Weaverdyck & Parkinson, 2018). Knowing that a stranger has a trustworthy friend may increase the likelihood that you would trust this stranger to watch your laptop. In other words, we may believe that a stranger will behave similarly to how their friend behaves due to an assumption of similarity among friends.

It is also unclear how third-party relationship knowledge (i.e., knowledge of others’ relationships) affects our perceptions of someone we *have* previously encountered (i.e., people about whom we have direct knowledge). When encountering people we know (e.g., acquaintances and friends), perceivers spontaneously retrieve information about those people’s relationships, presumably to prepare for appropriate, beneficial interactions (Parkinson et al., 2017). Thus, it is possible that people use information about others’ relationships when deciding how to behave in an upcoming interaction. For instance, if a recent acquaintance has consistently behaved trustworthily, but you know they have a deceitful friend, you may be less trusting of that individual, compared with another recent acquaintance who behaves identically but has friends whom you trust. Thus, when interacting with a familiar other, people may continue to refer to their knowledge of that individual’s relationships.

## Do People Expect Those Who Behave Similarly to Be Friends?

While beliefs about a partner’s relationships may shape predictions about how that partner will behave in an upcoming interaction, others’ behavioral tendencies (i.e., how they typically behave in particular situations) may also shape our beliefs about those individuals’ relationships with one another. In human social networks, friendship is linked to similarity in a wide variety of factors, including some forms of prosocial behavioral tendencies (Apicella et al., 2012). Thus, people may expect their interaction partners to be friends with others who think and behave like they do, and accordingly, this expectation may shape mental representations of social networks. For example, you may be unsurprised to learn that two people who both value honesty or rule-following are friends with one another, and you may even *expect* or *assume* that these two people would be friends with each other if you learned that they both belonged to the same small community. In the same vein, you may also expect two people within that community who are both rebellious to be friends with one another. On the contrary, you may be surprised to learn that an exceptionally rebellious person is friends with someone who is a stickler for the rules, or that an honest, trustworthy person is friends with someone who is conniving and deceitful. More generally, if two individuals tend to behave similarly, people may be more likely to believe that those individuals are friends with one another, even when they are not, compared with a pair of individuals who behave dissimilarly to one another.

## This Study

In this study, we sought to test how knowledge of others’ social relationships shapes people’s subjective expectations about how those individuals will behave, both when interacting with strangers and when repeatedly interacting with the same people. In addition, we examined how knowledge of others’ behavior shapes beliefs about friendship ties. That is, we also tested whether people are more likely to assume that others are friends^1^ with one another if they behave similarly to each other. Thus, rather than testing what is true (e.g., whether or not friends behave exceptionally similarly to each other), in this study, we test what people *assume* to be true (e.g., whether or not people believe that friends will behave exceptionally similarly to each other) and the consequences of such assumptions for social interactions and memory.

**Figure 1.**
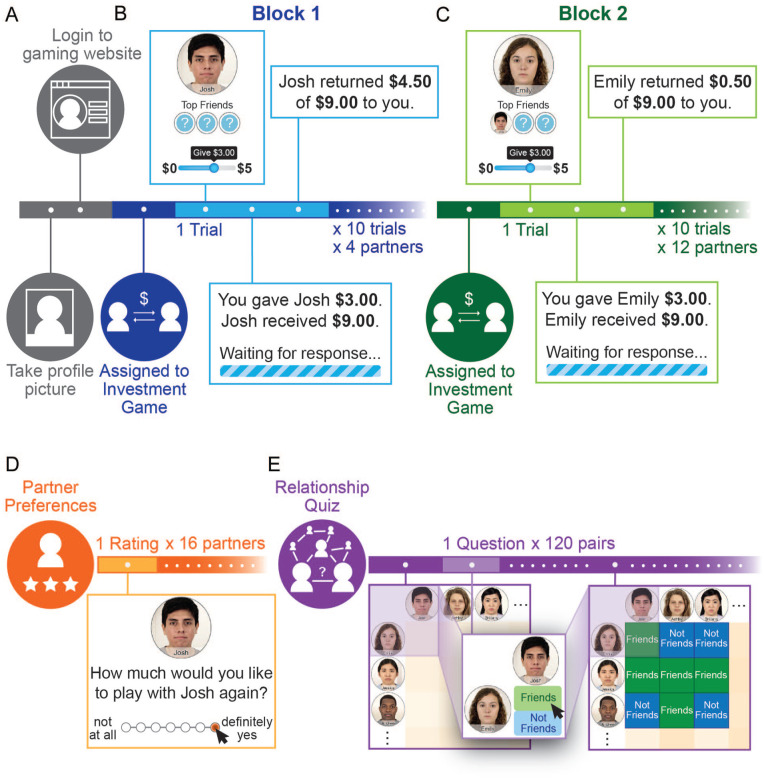
Overview of Paradigm. *Note.* Participants were told that they would be testing out a new online gaming community in which members had profiles and regularly played a variety of games with one another. As shown in (A), the experimenter first created a profile for the participant. Next (B), participants were “randomly assigned” to play a series of 10 investment games (i.e., trust games) with each of four partners. When playing with each partner, that individual’s name and profile picture were shown, along with their “Top 3 Friends” in the community, who were hidden if the participant had not already “met” them on the website, ostensibly due to the website’s privacy controls (see Figure 2). Thus, in Block 1 (B), the identities of participants’ partners’ “Top 3 Friends” were hidden. In Block 2 (C), participants were assigned to play the investment game again with 12 new partners, some of whom were indicated to have a “Top Friend” with whom the participant had interacted during Block 1, and thus, whose identity was visible. Afterward (D), participants rated each partner on how much they would like to play with them again in a new, cooperative game. Finally, participants completed a surprise memory test (E) of who appeared as friends and who did not by filling out a matrix with “Friends” or “Not Friends” for every pair of partners.

**Figure 2.**
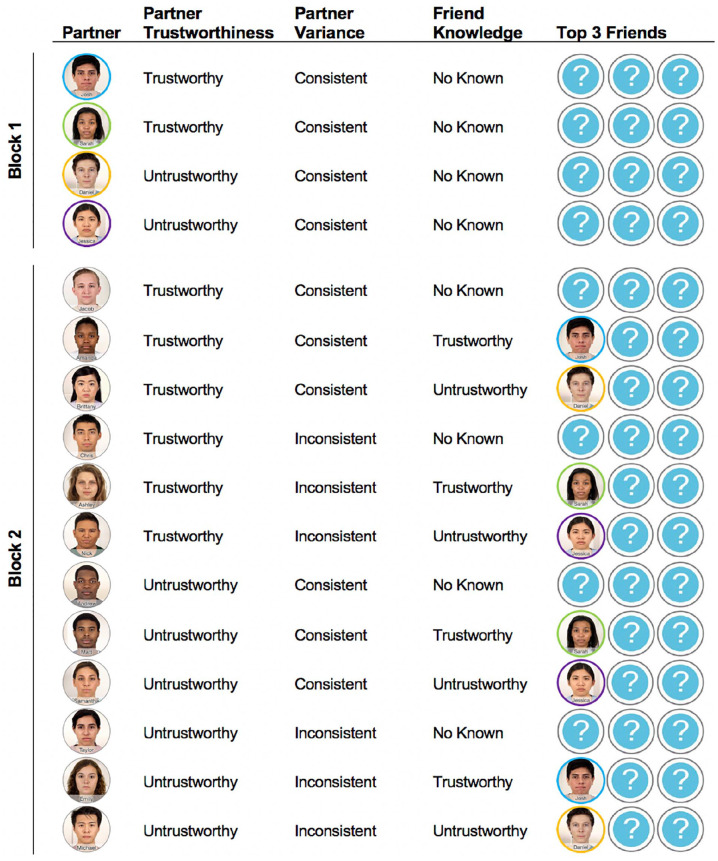
Partner Characteristics. *Note.* Participants played repeated trust games with four partners in Block 1 (top) and 12 partners in Block 2 (bottom). *Partner*: Each partner was identified with a profile picture and name. *Partner Trustworthiness*: Each partner’s reciprocation rate was drawn from a Gaussian distribution that had a mean of either 50% (for “trustworthy” partners) or 5% (for “untrustworthy” partners). *Partner Variance*: The distribution from which a partner’s reciprocation rate was drawn on each trial had a variance of either 0.01 (for “consistent” partners) or 0.12 (for “inconsistent” partners). *Top 3 Friends*: If the participant had already played with a partner’s friend, then they would be able to see who that friend was. If they had not played with a partner’s friend yet, then that friend was kept anonymous and only a question mark was shown. Participants did not know any of their partners’ friends in Block 1. In Block 2, participants knew either zero or one of each partner’s friends. *Friend Knowledge*: Participants either did not know any of the partner’s friends (“no known”), or they knew that they had a trustworthy friend (“trustworthy”) or untrustworthy friend (“untrustworthy”) whom they had played in Block 1 (images with colored borders). In Block 2, there was one partner for each cell of the 2 × 2 × 3 factorial design. Images and names were randomly assigned to partners/conditions for each participant. Note: Colored borders are only shown here to clearly depict correspondences between Block 1 partners and Block 2 partners’ “Top 3 Friends,” and did not appear in the experiment.

To this end, we constructed a fictive online game-playing community in which participants played games with various partners (see Figures 1 and 2). By creating this fictive online game-playing community, we were able to measure and manipulate participants’ beliefs about both their gaming partners’ behavior and the friendships between those partners in a fully balanced design. We thus examined how beliefs about others’ behavioral tendencies shape, and are shaped by, beliefs about their relationship ties. More specifically, we tested two complementary hypotheses: Assumptions of similarity among friends will lead people to expect partners who are friends to behave similarly (Hypothesis 1), and assumptions of similarity among friends will lead people to expect that two partners are more likely to be friends if they behave similarly than if they behave dissimilarly (Hypothesis 2).

## Methods

Materials and code used to conduct the experiment are publicly available (https://github.com/meng-du/TGame), as are all data and analysis scripts (https://osf.io/nx87u/).

## Participants

Participants were 80 undergraduate students (50 females, 29 males, and 1 did not specify) at the University of California, Los Angeles, between 18 and 31 years old (*M* = 20.51 years, *SD* = 2.37). Two additional participants were excluded from analyses because, after finishing the experiment, they explicitly told the experimenter that they either did not understand the instructions or fell asleep during the experiment. All measures, manipulations, and exclusions are reported in this article. Based on the medium-to-large effect sizes found in studies utilizing similar paradigms (Chang et al., 2010; van’t Wout & Sanfey, 2008), we determined that we would need 63 participants to detect the smallest of these effects (η^2^*=* 0.05; Chang et al., 2010) with 80% power. These calculations were made in the R package pwr (Champely, 2018) and were based on detecting a main effect of friend knowledge in a balanced one-way analysis of variance (ANOVA) (although a more appropriate linear mixed effects model was implemented). Sample size was determined before any data analysis. All participants provided written informed consent in accordance with the policies of the University of California, Los Angeles ethical review board.

## Procedure

### Introduction to the Online Game-Playing Community

Before the study began, participants were told that the experimenter needed to make profiles for them, so that they could join an online game-playing community. The experimenter took a facial photograph of the participant (Figure 1A). The photograph would ostensibly serve as the participant’s profile picture on the website, which would be displayed to partners during game play (participants later saw analogous photographs of their partners when interacting with them). Participants were told that this online game-playing community was being developed and tested at multiple college campuses, and that members can win small amounts of money by playing a variety of simple interactive games. Specifically, participants were informed that they would receive a portion of the total money that they earned while playing on the site. They were also told that their partners would be players from other campuses who regularly engage with the website and its associated social networking features; these individuals had ostensibly played a variety of games on the website several times before and continue to do so regularly for fun and profit. In reality, participants were playing with a computer that simulated different behavioral tendencies (see “Manipulating Interaction Partners’ Behavior” section). In addition, participants were told that the gaming website was also rolling out a new social network feature, in which each player’s “Top 3 Friends” (described in the “Manipulating Social Network Knowledge” section) would be displayed along with their profile photograph. Deception was used in order to manipulate our key constructs of interest and to maximize the equivalence in experience across participants. Participants were fully debriefed regarding all aspects of the procedure immediately after finishing the study.

Next, participants were told that they would be randomly assigned to play one game out of a set of many possible games during each of three consecutive blocks. In reality, all participants were assigned to play trust games (described in the “Manipulating Interaction Partners’ Behavior” section; Berg et al., 1995) in the first two blocks. Participants were then told that the third block would be skipped due to time constraints. This was done to further instill the idea that members of the website (including the participant) could have played a variety of games, and to make participants believe, during the second block, that they might not be done with interacting with their partners just yet. The trust game was always referred to as “The Investment Game” to minimize demand characteristics. As motivation, participants were informed that they would be paid at the end of the experiment based on their earnings during the game. Participants were also informed that as a member of this website, they would later have the chance to rate each partner, and that these ratings contribute to the determination of each member’s “Top 3 Friends,” as described in more detail in the next section.

### Manipulating Social Network Knowledge

To test whether friendship knowledge shapes how participants expected people to behave (Hypothesis 1), we systematically manipulated friendships between partners. For each interaction, participants viewed their partners’ first names and profile photographs, along with photographs of their partners’ “Top 3 Friends” (Figure 1B and 1C). Prior to the start of the experiment, it was explained that members regularly answered questions about how much they favored one another and how much they would like to engage with one another in the future; an algorithm allegedly computed each member’s “Top 3 Friends” based on who that member consistently favored and was favored by. Participants were also told that, due to the default privacy settings on the website, the faces of a given partner’s “Top 3 Friends” would not be visible if the participant had not previously played a game with that friend on the website. Instead, question marks would be displayed in place of those individuals’ faces (Figures 1B, 2). Thus, participants were unable to view any of their partners’ friends in Block 1 (Figure 2), but were then able to see the face and name of up to one friend for Block 2 partners (i.e., friends of Block 2 partners who were people the participant had encountered in Block 1; Figure 2). Half of the Block 2 partners’ visible friends were known by the participant to be trustworthy (based on the Block 1 games), and half were known to be untrustworthy. For a full description of all partners’ relationships and behavioral tendencies, see Figure 2.

### Manipulating Interaction Partners’ Behavior

In each round of a trust game, the participant is endowed with a sum of money (US$5 in this study). They must choose a portion of that money for the experimenter to triple and send to their partner, who can then choose to return any amount of the tripled sum to the participant (Figure 1B and C; Berg et al., 1995). It is maximally advantageous for the participant to invest all of their endowment if their current partner can be trusted to return more than one third of what they receive; investing in an untrustworthy partner (i.e., someone who returns less than one third of the tripled sum), however, leads to a net loss. Participants always played the role of the initial allocator (Player 1) in the trust games, and never the role of the player who received the initial offers (Player 2).

In this study, unbeknownst to participants, a computer algorithm determined the average proportion of the tripled sum each of their partners would return to them on each trial (i.e., their trustworthiness). Trustworthy partners’ return rates on each trial were drawn from a Gaussian distribution with a mean of 50%, whereas untrustworthy partners’ return rates were drawn from a Gaussian distribution with a mean of 5%. Furthermore, some partners were consistent (their return rates were drawn from a Gaussian distribution with a relatively low variance of 0.01), whereas others were inconsistent (their return rates were drawn from a Gaussian distribution with a relatively high variance of 0.12). This offer variance was used for exploratory analyses examining how the relative consistency of a social partner’s behavior impacts the extent to which one relies on knowledge of that partner’s friends when forming an impression of them. In addition to manipulating partner trustworthiness (two levels: untrustworthy and trustworthy) and the variance of their offers (two levels: inconsistent and consistent), we also controlled participant’s friend knowledge (i.e., apparent friendships between partners) as described above (three levels: untrustworthy friend, trustworthy friend, and no known friends).

This paradigm provides a quantifiable behavioral measure of how much an individual trusts their partner, while allowing the experimenter to maintain experimental control over the partners’ behavior and apparent relationships. By manipulating partners’ return rates and known associates, it is possible to measure how knowledge gained through direct experiences with a partner and knowledge of that partner’s relationships shape interpersonal trust, as well as how partners’ behaviors shape beliefs about their relationships. That is, the current experimental approach (a) allows us to decouple social behavioral tendencies from other variables that often covary in real-world situations, (b) ensures that we have data in each cell of the experimental design (e.g., trustworthy partners with no known, trustworthy, and untrustworthy friends) to allow us to test our hypotheses (e.g., to test how first interactions between partners with untrustworthy friends differ from those with trustworthy or no known friends), and (c) provides a strong test of our hypotheses by introducing social partners with precisely equivalent behavioral tendencies paired with differing friendship information and testing for the impact of that friendship information on future partner preferences and interactions within the trust game.

#### Block 1 of Trust Games

In the first block, participants played with two consistently trustworthy partners and two consistently untrustworthy partners (all Block 1 partners behaved in a consistent manner to facilitate learning; Figure 2). Participants played 10 rounds of the trust game with each of their Block 1 partners in a random, interleaved order, allowing them to learn the relative trustworthiness of each of these four individuals (Chang et al., 2010; Fareri et al., 2012, 2015).

#### Block 2 of Trust Games

In Block 2, participants played with 12 new partners. As in Block 1, participants played 10 rounds of the trust game with each partner in a random, interleaved order. Participants’ Block 2 partners varied on all three possible dimensions: partner trustworthiness, variance, and apparent friendships (as described above). Of the 12 partners in Block 2, there were six trustworthy and six untrustworthy partners, six consistent and six inconsistent partners, and four partners each with no known, trustworthy, and untrustworthy friends. Thus, each partner filled a different cell of the 2 × 2 × 3 factorial design (Figure 2).

#### Stimuli

Great care was taken to make the online game-playing experience as naturalistic as possible. This was done to ensure that participants believed they were playing with human partners, while still maintaining sufficient experimental control to be able to manipulate participants’ beliefs about their partners’ behavior, and the friendships between those partners, in a fully balanced design (which allowed us to systematically test the relationships between these variables). On each trial, participants saw their partner’s picture with at most one other picture of that partner’s top friends. The 16 photographs (see Figure 2) were selected from the Chicago Face Database (Ma et al., 2015) and consisted of neutral photographs of male and female Asian, Black, Latinx, and White individuals. The images were altered to look more naturalistic (e.g., partners appeared to be wearing various colored shirts in front of natural white walls, rather than the same gray t-shirt in front of artificially removed backgrounds), and their ostensible first name was overlaid on the photograph (Figure 2). Below the images was a slider from US$0 to US$5 which the participant used to make their offers (Figure 1B and 1C). After making each offer, a progress bar appeared as the partner was ostensibly making their decision. For each participant, the partner photographs and names were randomly assigned to partner condition (i.e., consistent/inconsistent behavior, trustworthy/untrustworthy behavior, and trustworthy/untrustworthy/no known friends). This was done to ensure that there would be no systematic relationships between the behavioral tendencies of participants’ partners and aspects of those partners’ physical appearance (e.g., race, gender, apparent trustworthiness, and attractiveness).

### Measuring Generalized Partner Preferences

As described above, the amount that a participant offered on each round of the trust game provided a measure of how much that participant trusted that partner, which, in turn, provided a way to test whether participants expected friends to behave similarly (Hypothesis 1). To further test whether participants expected friends to behave similarly beyond the immediate context, we measured their general preferences for playing with each partner. After the two blocks of trust games, participants rated each partner from both blocks in terms of how much they would like to play with them again in a different context in the future by responding to the following prompt (adapted from Hackel et al. (2015)): ‘‘You may have the chance to be invited back to complete a cooperative puzzle-solving game with a partner. If this happens, we’ll do our best to follow your preferences in assigning you a partner. Please rate how much you would like to be paired with each partner you played with today’’ (Figure 1D). Responses to this question provided a measure of participants’ more general preferences for engaging with each partner in the future, beyond the immediate context of the trust game.

### Measuring Perceived Friendships Between Interaction Partners

To test whether participants expected similarly behaving partners to be friends (Hypothesis 2), participants completed an incidental memory test assessing their knowledge of their partners’ social network at the end of the study. Participants saw a grid with all 16 partners’ profile pictures along the rows and columns (Figure 1D). They responded to the prompt, “For each pair of players, please indicate whether they appeared as friends in your previous games.” This was done by selecting either the label “Friends” or “Not Friends” in each cell of the matrix, indicating whether the participant thought the partners in the corresponding row and column were in each other’s “Top 3 Friends.”

At the end of the experiment, participants provided basic demographic information (age, gender, ethnicity) and feedback regarding their enjoyment of the game to maintain the cover story regarding the purpose of the experiment. All tasks were completed alone in a private room.

## Analyses

Analyses of all data were implemented in R (version 3.6.1). Linear mixed models were implemented using the package nlme (Pinheiro et al., 2019). All means and SEs reported for linear mixed models are estimated marginal means (i.e., least-squares means) and SEs using the package emmeans (Lenth, 2019). The reported *p* values for all pairwise *t*-tests have been corrected for multiple comparisons using the Holm correction (Holm, 1979).

## Results

## Do People Expect Friends to Behave Similarly? (Testing Hypothesis 1)

Block 1 was used for the sole purpose of manipulating participants’ beliefs about the trustworthiness of future partners’ friends. As such, only offers made in Block 2 were used to test the main hypotheses of this study. Please refer to the Supplemental Material for a comprehensive analysis of participants’ behavior during Block 1 (Figure S1, Table S1).

**Table 1.** Linear Mixed Model on Block 2 Offers Over Time.

Effect	β	95% CI	*F*	*DF1*	*DF2*	*p*
Partner Trustworthiness	0.79	[0.67, 0.90]	192.55	1	9,485	<.001***
Friend Knowledge			9.10	2	9,485	<.001***
Trustworthy—No Known Friends	0.02	[−0.03, 0.07]				
Untrustworthy—No Known Friends	−0.11	[−0.16,−0.05]				
Variance	0.08	[0.04, 0.12]	17.72	1	9,485	<.001***
Trial	−0.04	[−0.07, −0.02]	13.21	1	9,485	<.001***
Trial^2^	0.02	[0.01, 0.02]	91.61	1	9,485	<.001***
Partner Trustworthiness: Friend Knowledge			6.63	2	9,485	.001**
Trustworthy—No Known Friends	−0.02	[−0.07, 0.03]				
Untrustworthy—No Known Friends	−0.07	[−0.12, −0.02]				
Partner Trustworthiness: Variance	0.16	[0.12, 0.19]	71.21	1	9,485	<.001***
Friend Knowledge: Variance			4.52	2	9,485	.011*
Trustworthy—No Known Friends	0.02	[−0.03, 0.07]				
Untrustworthy—No Known Friends	0.06	[0.00, 0.11]				
Partner Trustworthiness: Trial	0.08	[0.06, 0.09]	93.39	1	9,485	<.001***
Partner Trustworthiness: Trial^2^	−0.02	[−0.02, −0.01]	93.71	1	9,485	<.001***
Friend Knowledge: Trial			3.58	2	9,485	.028*
Trustworthy—No Known Friends	−0.01	[−0.02, 0.00]				
Untrustworthy—No Known Friends	0.02	[0.00, 0.03]				
Friend Knowledge: Trial^2^			1.56	2	9,485	.210
Trustworthy—No Known Friends	0.00	[0.00, 0.01]				
Untrustworthy—No Known Friends	0.00	[−0.01, 0.00]				
Variance: Trial	0.02	[0.01, 0.03]	15.55	1	9,485	<.001***
Variance: Trial^2^	0.00	[−0.01, 0.00]	1.29	1	9,485	.256
Partner Trustworthiness: Friend Knowledge: Variance			5.11	2	9,485	.006**
Trustworthy—No Known Friends	0.08	[0.03, 0.14]				
Untrustworthy—No Known Friends	−0.04	[−0.09, 0.01]				
Partner Trustworthiness: Friend Knowledge: Trial			2.38	2	9,485	.093^†^
Trustworthy—No Known Friends	0.00	[−0.01, 0.01]				
Untrustworthy—No Known Friends	−0.01	[−0.02, 0.00]				
Partner Trustworthiness: Friend Knowledge: Trial^2^			1.51	2	9,485	.220
Trustworthy—No Known Friends	0.00	[0.00, 0.01]				
Untrustworthy—No Known Friends	0.00	[0.00, 0.01]				
Partner Trustworthiness: Variance: Trial	0.02	[0.01, 0.03]	28.61	1	9,485	<.001***
Partner Trustworthiness: Variance: Trial^2^	0.00	[−0.01, 0.00]	3.18	1	9,485	.075^†^
Friend Knowledge: Variance: Trial			2.18	2	9,485	.113
Trustworthy—No Known Friends	0.01	[0.00, 0.02]				
Untrustworthy—No Known Friends	0.00	[−0.01, 0.01]				
Friend Knowledge: Variance: Trial^2^			0.15	2	9,485	.859
Trustworthy—No Known Friends	0.00	[0.00, 0.01]				
Untrustworthy—No Known Friends	0.00	[−0.01, 0.00]				
Partner Trustworthiness: Friend Knowledge: Variance: Trial			0.48	2	9,485	.622
Trustworthy—No Known Friends	0.00	[−0.01, 0.02]				
Untrustworthy—No Known Friends	0.00	[−0.01, 0.01]				
Partner Trustworthiness: Friend Knowledge: Variance: Trial^2^			1.80	2	9,485	.165
Trustworthy—No Known Friends	0.00	[−0.01, 0.00]				
Untrustworthy—No Known Friends	0.00	[0.00, 0.01]				

*Note.* Results from a linear mixed effects model with offer amount as the dependent variable, and friend knowledge, partner trustworthiness, partner variance, and both linear and quadratic transformations of time (centered Trial) as fixed effect predictors. Random intercepts and by-trial linear slopes were included for each participant for each level of trustworthiness. For effects including friend knowledge, which consists of three levels and two estimates, and CIs are provided. The first value reflects the difference between offers to partners with trustworthy friends and those with no known friends, and the second reflects the difference between offers made to partners with untrustworthy friends and those with no known friends. CI = confidence interval.

† *p* < .100; *p < .050; **p < .010; ***p < .001.

### Interacting With Partners for The First Time

**Figure 3.**
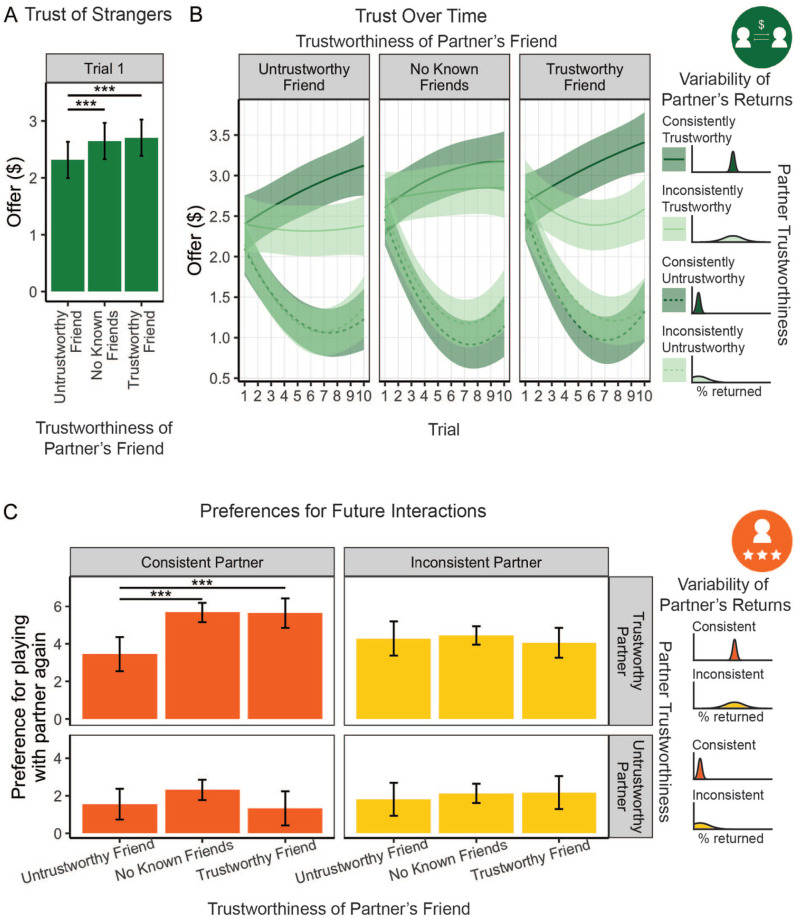
Knowing Who Is Friends With Whom Shapes Social Behavior. *Note.* In Block 2, (A) participants initially (i.e., before they had direct knowledge of each partner’s trustworthiness) offered smaller amounts to partners with untrustworthy friends than to those with either trustworthy or no known friends. Even after gaining direct knowledge of their partners’ behavior, (B) the same main effect of friend knowledge held, such that partners with untrustworthy friends were offered less than partners with no known or trustworthy friends. This main effect of friend knowledge appeared to be driven by cases where the partners themselves were trustworthy (top panel) and consistent (dark green), who were offered more and more over time. Participants were less willing to interact in a (C) new, cooperative context in the future with partners who had untrustworthy friends than those with either no known or trustworthy friends, but only if those partners were consistently trustworthy themselves. Trial variable was centered around 0 in the model and relabeled with 1 to 10 here for clarity. Asterisks indicate significant differences between friend knowledge conditions (***p < .001). Error bars show 95% CI.

Participants’ initial offers to their Block 2 partners provided a window into how third-party relationship knowledge may shape expectations about how strangers will behave. We ran a linear mixed effects model on offers made to each *new* partner in Block 2. Friend knowledge was included as a fixed effect predictor, along with random intercepts for each participant (which account for nonindependence among observations of the same participant). This analysis revealed a significant main effect of friend knowledge on participants’ initial trust in strangers, β_trustworthy-unknown_ = 0.15, 95% confidence interval (CI) = [0.08, 0.22], β_untrustworthy-unknown_ = −0.24, 95% CI = [−0.31, −0.17], *F*(2, 878) = 23.62, *p* < .001. Subsequent pairwise *t*-tests on the least-squares estimation of the means revealed that participants offered partners with untrustworthy friends (*M* = 2.32, *SE* = .16) significantly less than those with either trustworthy friends (*M* = 2.71, *SE* = .16), Δ = 0.39, 95% CI = [0.24, 0.54], *t*(878) = 6.37, *p* < .001, *d* = 0.43, or no known friends (*M* = 2.65, *SE* = .16), Δ = 0.33, 95% CI = [0.19, 0.48], *t*(878) = 5.42, *p* < .001, *d* = 0.37 (Figure 3A). However, there was no significant difference between offers made to partners with trustworthy friends and those with no known friends, Δ = 0.06, 95% CI = [−0.09, 0.21], *t*(878) = 0.95, *p* = .343, *d* = 0.06. The pattern of significance of these effects was not impacted by taking into account other extraneous factors that could shape participants’ initial interactions with new partners, such as whether those partners belonged to the same or different demographic categories than the participant (see Supplemental Material).

These findings support our first hypothesis, as they suggest that participants were wary of new partners who had untrustworthy friends, deciding to risk less in those encounters than when playing with partners who had trustworthy friends or unknown friends. Interestingly, it did not matter whether the participant knew nothing about a partner’s friends (i.e., no known friends) or if they knew they were trustworthy. If we consider *not* knowing who someone’s friends are as the baseline, then it appears that having trustworthy friends provided no benefit in new encounters. However, having an untrustworthy friend resulted in less trust being bestowed.

### Repeatedly Interacting With Partners

Next, we tested if and how participants’ beliefs about their partners’ trustworthiness were shaped by (a) knowledge of their partners’ social relationships and (b) directly acquired knowledge of their partners’ behavioral tendencies. We examined how friend knowledge affected participants’ offers to their partners over time and their preferences for playing with those partners again in the future in different contexts.

#### Trust game offers

We again used a linear mixed effects model to test how offers were affected by friend knowledge (Table 1). This method was chosen because it accounts for several sources of nonindependence among observations. Specifically, this method accounts for the fact that participants may differ in the overall amounts that they tend to invest in partners who behave in a trustworthy or untrustworthy manner, and in the rate at which those offers change over time, through the inclusion of random by-participant intercepts and slopes, as described below. Furthermore, this approach also allows us to account for temporal autocorrelation by specifying a first-order autoregressive (AR1) covariance structure reflecting that correlations between offers made by a participant decrease as they become farther removed from one another in time. We included the linear effect of time (centered at 0) and its quadratic transformation to account for the possibility of a nonlinear trend of offers made over time. In total, we included the following fixed effects in this model: friend knowledge, partner trustworthiness, partner variance, and linear and quadratic transformations of time, along with all of their interactions. We also included random intercepts and linear slopes per participant for each level of partner trustworthiness. There was a significant main effect of friend knowledge (Table 1, Figure 3B), suggesting that participants continued to use information about their partners’ friendships even after having directly acquired information on which to draw. In addition, there was a significant interaction effect with the linear transformation of time. This suggests that knowledge of how someone’s friend tends to behave (i.e., their friend’s trustworthiness) affected how participants learned about that partner’s own behavioral tendencies (i.e., their own trustworthiness).

To better understand the three-way interaction effect of friend knowledge, partner trustworthiness, and variance (see Table 1), we ran pairwise *t*-tests on the estimated marginal means. That is, we compared each of the three levels of friend knowledge at each of the combined levels of partner trustworthiness and variance. This approach showed that the above effects were mostly driven by trustworthy partners, as described in more detail below. The only significant pairwise comparison for untrustworthy partners involved *inconsistently* untrustworthy partners; participants offered inconsistently untrustworthy partners who had trustworthy friends (*M* = 1.26, *SE* = .14) more than those who had untrustworthy friends (*M =* 1.13; *SE =* .14), Δ = 0.23, 95% CI = [0.01, 0.45], *t*(9,485) = 2.50, *p* = .037, *d* = 0.05. When a participant’s direct experience with a partner showed that they were *consistently* not to be trusted, however, that participant did not consider how trustworthy their friends were when deciding whether or not to trust them.

For trustworthy partners, on the other hand, there was a significant difference between partners who acted in a consistent way across trials (i.e., low variance) and those who behaved inconsistently across trials (i.e., high variance). When a partner was consistently trustworthy, the effects of friend knowledge were exactly the same as what was found with the Trial 1 data, supporting Hypothesis 1: Participants offered significantly less to those with untrustworthy friends (*M* = 2.83, *SE* = .16) than those with either trustworthy friends (*M* = 3.09, *SE* = .16), Δ = 0.26, 95% CI = [0.04, 0.48], *t*(9,485) = 2.85, *p* = .013, *d* = 0.06, or no known friends (*M* = 3.04, *SE* = .16), Δ = 0.21, 95% CI = [0.00, 0.44], *t*(9,485) = 2.32, *p* = .041, *d* = 0.05. This can be seen clearly in Figure 3B, where consistent, trustworthy partners (top panel, dark green lines) have similar slopes, irrespective of participants’ knowledge about their friends, but show the lowest intercept when they have an untrustworthy friend, rather than a trustworthy friend, or no known friends.

If a trustworthy partner was inconsistent in their return rates, however, participants offered those with no known friends (*M* = 2.81, *SE* = .16) considerably more than those with either trustworthy (*M* = 2.40, *SE* = .16), Δ = 0.41, 95% CI = [0.19, 0.63], *t*(9,485) = 4.53, *p* < .001, *d* = 0.09, or untrustworthy friends, (*M* = 2.32, *SE* = .16), Δ = 0.50, 95% CI = [0.28, 0.72], *t*(9,485) = 5.45, *p* < .001, *d* = 0.11 (Figure 3B, top panel, light green; see Supplemental Material for further analysis and discussion on how variability in partners’ returns modulates the use of friendship knowledge). Together, these results suggest that people were wary of potentially negative interactions: If their partner behaved in an untrustworthy way, or if they had untrustworthy friends, they were offered less money. If, however, they tended to be trustworthy and they had a trustworthy friend, but they were inconsistent (e.g., sometimes they returned much less than what was expected for a trustworthy partner with a trustworthy friend), then people trusted them significantly less than they would have if they did not have these priors (i.e., no known friends). For further analyses and discussion of how behavioral variability may influence how and when people use third-party knowledge, see the Supplemental Material (Figure S2).

#### Generalized Partner Preferences

After the game-playing portion of the experiment had concluded, participants rated how much they would like to play with each partner in a cooperative, and thus new context in the future. Since it is possible that participants did not accurately remember who was friends with whom, we tested how participant’s *true* knowledge about their Block 2 partners’ relationships affected their expectations of how those partners would behave in the future. We used the results of the incidental memory test participants completed at the very end of the experiment to only select rating data for partners whose relationships were correctly recalled. We then used a linear mixed model to test the effects of friend knowledge, partner trustworthiness, and partner variance on partner preference ratings (Table 2). The model included friend knowledge, partner trustworthiness, and partner variance as fixed effects, as well as random by-participant intercepts. Here, we saw similar results to what we found in the trust game data. There was a significant three-way interaction, which appeared to be driven by consistent, trustworthy partners who were rated lower if they had an untrustworthy friend (*M* = 3.45, *SE =* .46) than if they had a trustworthy friend (*M* = 5.64, *SE* = .40), Δ = 2.20, 95% CI = [0.74, 3.65], *t*(206) = 3.63, *p =* .001, *d* = 0.51, or no known friends (*M* = 5.68, *SE =* .27), Δ = 2.23, 95% CI = [0.96, 3.50], *t*(206) = 4.24, *p <* .001, *d* = 0.59. No other pairwise comparisons of friend knowledge were significant. In other words, participants’ ratings of their partners suggest that people used relationship knowledge when considering future interactions with partners who had behaved in a consistently trustworthy way, but not when considering future interactions with other partners (Figure 3C). Again, this suggests that participants did not trust people who acted in an untrustworthy manner, no matter who their friends were, and that they were wary of people who seemed trustworthy, but had untrustworthy friends.

**Table 2.** Linear Mixed Model on Preferences for Future Interactions With Partners.

Effect	β	95% CI	*F*	*DF1*	*DF2*	*p*
Friend Knowledge			5.82	2	206	.003**
Trustworthy—No Known Friends	0.06	[−0.26, 0.39]				
Untrustworthy—No Known Friends	−0.46	[−0.80, −0.13]				
Partner Trustworthiness	1.35	[1.14, 1.57]	150.59	1	206	<.001***
Partner Variance	0.09	[−0.13, 0.31]	0.66	1	206	.417
Friend Knowledge: Partner Trustworthiness			1.23	2	206	.296
Trustworthy—No Known Friends	0.20	[−0.13, 0.52]				
Untrustworthy—No Known Friends	−0.26	[−0.59, 0.07]				
Friend Knowledge: Variance			3.04	2	206	.050*
Trustworthy—No Known Friends	0.10	[−0.23, 0.42]				
Untrustworthy—No Known Friends	−0.36	[−0.69, −0.03]				
Partner Trustworthiness: Variance	0.24	[0.02, 0.46]	4.80	1	206	.030*
Friend Knowledge: Partner Trustworthiness: Variance			3.06	2	206	.049*
Trustworthy—No Known Friends	0.37	[0.04, 0.69]				
Untrustworthy—No Known Friends	−0.39	[−0.72, −0.06]				

*Note.* Results from a linear mixed model on participants’ preferences for interacting with partners again in a new context. Friend knowledge, partner trustworthiness, and partner variance were included as fixed effects, and random by-participant intercepts were also included in the model. Follow-up tests show that friend knowledge is particularly predictive of preferences when partners behave in a consistent and trustworthy manner. For effects including friend knowledge, which consists of three levels, two estimates and CIs are provided. The first value reflects the difference between ratings of partners with trustworthy friends and those with no known friends, and the second reflects the difference between preferences for partners with untrustworthy friends and those with no known friends. CI = confidence interval.

† *p* < .100; *p < .050; **p < .010; ***p < .001.

### Summary

Analyses of participants’ trust game offers and postgame preference ratings suggest that the assumption of similarity among friends shapes expectations about how others will behave (supporting Hypothesis 1). When initially interacting with strangers, people shape their behavior based on knowledge of how those individuals’ friends behave. Furthermore, in some cases (e.g., when a trustworthy-seeming individual is known to have untrustworthy friends), social network knowledge continues to shape expectations about a partner’s behavior, such that people remain wary of those who associate with untrustworthy people, even in the presence of countervailing evidence about that individual’s own behavior.

## Do People Expect Those Who Behave Similarly to Be Friends (Testing Hypothesis 2)?

The results in the previous section suggest that assumptions of similarity among friends lead knowledge of others’ social relationships to shape expectations about how they will behave. Next, we tested whether the converse would also be true. In other words, we tested whether knowledge of others’ behavior would shape how participants recall their social relationships. Specifically, we tested whether participants would expect similarly behaving individuals in a community to be friends with one another, even when they are not.

To test the effect of behavioral similarity on beliefs about friendship ties, we created two dyad-level variables to describe each pair of partners that participants were quizzed on (i.e., all possible unique pairs). The first such variable, dyad variance, had three levels: dyads composed of (a) one consistent and one inconsistent partner, (b) two consistent partners, or (c) two inconsistent partners. The second dyad-level variable, dyad trustworthiness, had two levels: each pair consisted of two partners who either (a) behaved similarly (i.e., both trustworthy partners or both untrustworthy partners) or (b) behaved differently (i.e., one trustworthy partner and one untrustworthy partner). Consistent with other work investigating how schemas influence memory in nonsocial domains (Brewer & Treyens, 1981; De Brigard et al., 2017; Lampinen et al., 2001), we examined false alarms in the relationship quiz. Specifically, we calculated each participant’s false-positive rate for each type of dyad. False-positive rate refers to the number of partner pairs that a participant reported as “Top 3 Friends” who were never presented as such, compared with the total number of pairs who were never presented as friends:

$$False\;Positive\;Rate=\frac{\begin{array}{l} Number\;of\;pairs\;falsely\\ remembered\;as\;friends \end{array}}{\begin{array}{l} Total\;number\;of\;pairs\;who\\ are\;not\;friends \end{array}}$$

We used a linear mixed model to test the effects of dyad variance similarity (three levels: two consistent partners; two inconsistent partners; and one consistent and one inconsistent partner) and dyad trustworthiness similarity (two levels: similar overall trustworthiness and dissimilar overall trustworthiness) on false-positive rate in the friendship memory quiz. The model included the dyad partners’ variance similarity and trustworthiness similarity as fixed effects, as well as random intercepts for each participant. Because the data were bounded by 0 and 1, false-positive rates were arcsine transformed before being entered into the model (Freeman & Tukey, 1950). There were significant main effects of both dyad trustworthiness similarity, β = 0.03, 95% CI = [0.01, 0.04], *F*(1, 395) = 10.39, *p* = .001, and dyad variance similarity, β_consistent-mixed_ = 0.04, 95% CI = [0.01, 0.04], β_inconsistent-mixed_ = −0.07, 95% CI = [−0.09, −0.04], *F*(2, 395) = 16.18, *p* < .001, as well as their interaction, β_consistent-mixed_ = −0.04, 95% CI = [−0.06, −0.01], β_inconsistent-mixed_ = 0.02, 95% CI = [0.00, 0.04], *F*(2, 395) = 4.52, *p* = .011. This was also true when we included a covariate indicating whether or not two partners were of the same race and another accounting for shared gender between partners, β_trustworthiness_ = 0.02, 95% CI = [0.01, 0.04], *F*(1, 1645) = 9.05, *p* = .003; β_consistent-mixed_ = 0.03, 95% CI = [0.01, 0.05], β_inconsistent-mixed_ = −0.06, 95% CI = [−0.08, −0.04], *F_variance_*(2, 1645) = 12.81, *p* < .001; β_consistent-mixed_ = −0.03, 95% CI = [−0.06, −0.01], β_inconsistent-mixed_ = 0.04, 95% CI = [0.02, 0.06], *F_interaction_*(2, 1645) = 6.87, *p* = .001.

**Figure 4.**
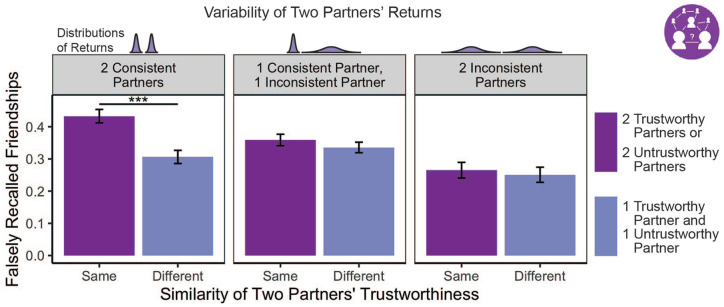
Behavioral Similarity Shapes Beliefs About Who Is Friends With Whom *Note.* Participants were more likely to falsely believe that two partners who behaved similarly (i.e., both trustworthy or both untrustworthy) were friends than two partners who behaved *dissimilarly* (i.e., one trustworthy and one untrustworthy). However, this was only true if both partners behaved in a consistent manner (i.e., both low variance). Note, for clarity, only the significance of effects of central interest to this study (i.e., the effects of friend knowledge on participants’ behavior) is indicated visually with asterisks (***p < .001). The *y*-axis reflects the arcsine transformation of false-positive rates. Error bars show 95% CI.

Follow-up paired *t*-tests on the estimated marginal means suggest that the main effect of dyad trustworthiness similarity was driven by consistently behaving pairs. This effect was significant when both partners behaved consistently (*M_similar_* = 0.43, *SE* = .04; *M_dissimilar_* = 0.31, *SE* = .04), Δ = 0.13, 95% CI = [0.07, 0.18], *t*(395) = 4.31, *p* < .001, *d* = 0.43, but not when both partners behaved inconsistently, or when one partner behaved in a consistent manner and the other did not (Figure 4). That is, participants were more likely to falsely remember a pair of partners as friends if they consistently behaved similarly to one another—that is, both trustworthy or both untrustworthy—supporting our second hypothesis that people who behave similarly are expected to be friends. This pattern of results also held when looking at trustworthy and untrustworthy pairs separately, such that there was no difference in false-positive rates between consistently trustworthy (*M* = .40, *SE* = .04) and untrustworthy pairs (*M* = .41, *SE* = .04), but both had significantly greater false-positive rates than pairs that behaved dissimilarly (*M* = .31, *SE* = .04), Δ_*trustworthy*_ = 0.09, 95% CI = [0.00, 0.17], *t*(632) = 2.53, *p* = .023, *d* = 0.20; Δ_*untrustworthy*_ = 0.11, 95% CI = [0.02, 0.19], *t*(632) = 2.98, *p* = .009, *d* = 0.24.

### Summary

Beliefs about others’ behavioral tendencies impact how people recall social relationships between others, such that individuals who behave similarly are more likely to be falsely remembered as friends (Hypothesis 2). This suggests that the assumption of similarity among friends shapes mental representations of social networks.

## Discussion

In real-world social networks, people tend to be socially connected with those who are similar to themselves. Here, we tested if and how assumptions of similarity among friends shape our beliefs about other people’s relationships and behavioral tendencies by having participants play a series of trust games in a fictive online community. More specifically, we tested two hypotheses: That assumptions of similarity among friends will lead people to expect partners who are friends to behave similarly (Hypothesis 1), and that assumptions of similarity among friends will lead people to expect that two partners are more likely to be friends if they behave similarly than dissimilarly (Hypothesis 2).

We frequently make judgments about how to interact with other people, including how much to trust them. Sometimes, we must decide whether or not to trust a complete stranger without any knowledge about that individual. Even in the absence of direct experience with someone, there is a wealth of information on which one can draw when making such decisions. One source of information is the web of relationships in which we, and all of our interactions, are embedded. Here, we found that knowledge of others’ social ties shapes evaluations of their behavioral tendencies. That is, people assumed similarity among friends, leading them to expect that partners who were friends with each other were more likely to behave similarly to each other (supporting Hypothesis 1) and that partners who behaved similarly were more likely to be friends (supporting Hypothesis 2).

## Social Network Knowledge Shapes Social Behavior

On the first encounter (i.e., first trust game trial) with each new partner, participants made trust decisions with no prior information about their partner’s own behavior. Using third-party relationship knowledge, participants offered less money to those with untrustworthy friends than to other partners, consistent with recent work demonstrating that knowledge of social affiliations biases decisions when interacting with strangers for the first time (Martinez et al., 2016). In this study, even after gaining direct experience with each partner, participants continued to partially rely on third-party relationship knowledge when making trust-based decisions, offering less money to partners with untrustworthy associates. Interestingly, this was only true when the third-party information signaled a potentially untrustworthy partner: In both the initial offers and subsequent trials, participants did not treat partners with trustworthy friends differently than those with no known friends, but consistently treated those with untrustworthy friends as less trustworthy. This pattern of results was also reflected in participants’ postgame preference ratings of each partner. People were significantly less inclined to cooperate in the future with partners who had untrustworthy friends than those with either trustworthy or no known friends. This suggests that people are especially avoidant of cues to potentially risky future social interactions. Taken together, these results provide convergent evidence that knowledge of a social partner’s relationships with others shapes one’s immediate and long-term expectations of that partner’s behaviors.

## Knowledge of Others’ Behavioral Tendencies Shapes Social Network Beliefs

We also found that a partner’s behavioral tendencies (i.e., their trustworthiness in the game) shaped participants’ perceptions of third-party ties. Specifically, we tested participants’ recall of each partner’s friendship ties at the end of the experiment and found that participants were more likely to misremember nonexistent friendships between partners who behaved similarly (i.e., both were predictably trustworthy or untrustworthy) compared with those who behaved dissimilarly. That is, knowledge of others’ behavioral tendencies shaped memories of their social ties.

Past research on the occurrence (McPherson et al., 2001), causes (Currarini et al., 2009), and assumption (Flynn et al., 2010) of similarity among friends has focused on relatively coarse variables (e.g., demographics). More recent research provides evidence for similarity among friends in terms of behavioral tendencies and how individuals process the world around them (Apicella et al., 2012; Centola, 2011; Parkinson et al., 2018; Pradel et al., 2009; K. M. Smith et al., 2018). The current results suggest that people may internalize such associations between behavioral similarity and social network proximity, and that this in turn may shape, and sometimes distort, their mental representations of social networks.

That said, it is important to note that the evidence for links between social affiliation and interpersonal similarity in cooperative behavior has been somewhat mixed (i.e., it is not always observed; Ehlert et al., 2020; Simpson et al., 2014; Vernarelli, 2016). Given that participants were led to believe that other players’ friendships with one another were based on numerous and diverse interactions on the social gaming website, it is possible that participants inferred that similarly behaving players had broader interpersonal similarities (e.g., in terms of their general underlying personality traits and values) that led them to both befriend one another and to behave similarly to one another when playing the investment game. It is possible that different results would be obtained (e.g., the assumption of similarity among friends may have been weaker) had participants been led to believe that their partners only interacted with one another in investment games, given that the link between similarity and social closeness in cooperative tendencies is not always observed. We suggest that future research examine whether the assumption of similarity among friends is applied uniformly across traits and contexts, or whether the existence and/or magnitude of this assumption, and its impact on people’s thoughts and behaviors, varies depending on the trait and/or context at hand (e.g., depending on the extent to which the assumption of similarity among friends reflects the ground truth for a particular attribute).

## Potential Mechanisms

Future work could build on the current findings by elucidating the underlying mechanisms that may give rise to people assuming a link between friendship and interpersonal similarity. It may be that people observe this association in their own social networks and thus assume it exists when interacting with new individuals. Indeed, homophily (i.e., the increased tendency for similar people to become socially linked compared with dissimilar individuals; Lazarsfeld & Merton, 1954) based on readily observable socio-demographic characteristics is commonly observed in real-world social networks. It may be that people who have similar cooperative tendencies are more likely to become friends, thus leading to this social prior. The well-documented effect of social contagion in cooperation (Christakis & Fowler, 2013; Fowler & Christakis, 2010) may also be a driving force in expectations of behavioral similarity. For instance, it could be that people believe partners with untrustworthy friends are more likely to become untrustworthy themselves over time—which could be addressed with future research on how people anticipate networks to form. Finally, there could also be biases in preferences and opportunities (Currarini et al., 2009), including similarity among socially close individuals that is driven by broader structural forces rather than solely by individual-level social choices (Schelling, 1978). Future research could integrate these lines of inquiry by examining if people who are more likely to interact with similar others in their real-world social networks also have a stronger tendency to expect people who are connected to one another to behave similarly, and vice versa. Similarly, we suggest that future research examine the match or mismatch between expectations and reality. More specifically, future research should test whether people’s expectations about how similarity among friends arises, along with the extent to which similarity is expected among friends, differ depending on (a) how likely that similarity is to exist in reality and (b) how much they themselves prefer to befriend similar others.

Prior work suggests that similarities among friends in their cooperative tendencies could arise through direct and indirect reciprocity (Chiang & Takahashi, 2011; Takahashi & Mashima, 2006), as well as mechanisms consistent with biological market theory (i.e., everyone prefers the best or most cooperative partners, resulting in each person being paired with others at their own level; Baumard et al., 2013; Chiang, 2010; Noë & Hammerstein, 1995). To decisively rule out the possibility that the current results reflect strategies related to indirect reciprocity or assumptions about biological market-related mechanisms, future work could extend the current experimental approach to similarity in attributes that are not inherently beneficial or detrimental to others (e.g., similarity in tastes or preferences, rather than trustworthiness). In addition, as previously noted, in this study, we were careful to present the gaming website and its members as an established community with a wide variety of ways of interacting with each other. The friendships viewed on this website, therefore, were ostensibly developed over time through a wide variety of games and interactions. This setup makes it somewhat unlikely that participants were assuming mechanisms such as reciprocity or biological markets: Two players who behaved in a selfish manner toward the participant in the investment game would not necessarily have regularly betrayed one another’s trust before (since their interactions could have been primarily in other kinds of game-playing contexts); furthermore, the qualities that make a desirable (or undesirable) partner in the investment game would not necessarily generalize to other games. Given that participants were led to believe that other players’ friendships were based on numerous and diverse interactions, similarly behaving people could have been expected to be friends with one another because broader similarities between them (e.g., in terms of their tastes, traits, and values) led both to friendship and to similar behavior within the context of the investment game or because broad interpersonal similarities among friends resulted from social influence. That said, we suggest that future work examine people’s beliefs about such phenomena (e.g., biological markets and reciprocity), the extent to which those beliefs reflect reality, and how such beliefs shape people’s expectations regarding how others will behave.

Rather than assuming similarity among friends, some of our results may reflect that participants expect relationships among three “nodes” in a triad to be balanced (Heider, 1958). In a triad where two nodes are people (e.g., the participant’s current partner and that partner’s friend) with a positive link between them (they like each other) and the third node is “being untrustworthy,” if the partner likes being untrustworthy, the triad is balanced if their friend does too. Thus, if a participant assumes that triads are balanced and knows about their current partner’s friend’s preference, they would assume that the partner would also prefer to be untrustworthy. Importantly, however, people do not always behave in ways consistent with their attitudes (one can regret a past behavior or dislike things about oneself), and we did not directly manipulate partners’ ostensible attitudes. Thus, further research is needed to delineate the mechanisms through which friendship knowledge impacts presumed behavioral similarities (and attitudes about such behaviors).

## Limitations and Future Directions

More generally, it remains to be seen how these results would generalize to other forms of social behavior (e.g., gossip), which may be affected differently by third-party ties due to social consequences (e.g., reputation management; Jolly & Chang, 2018; E. R. Smith & Collins, 2009). It is also unclear how these results would generalize across cultures. Given that homophily is thought to pervade social networks across diverse cultures and to have shaped the emergence and disappearance of ties in social networks from an early point in human history (Apicella et al., 2012), it may be the case that assumptions of similarity among friends serve as a social prior across cultures. That said, although the current study’s sample was ethnically diverse (see Supplemental Material S3), this research was limited to undergraduate students at a single American institution. The heuristics that guide social thought and behavior do not necessarily generalize across cultures; many psychological phenomena that were previously thought to generalize across cultures have since been shown to be specific to the population most often studied: people from western, educated, industrialized, rich, and democratic (WEIRD) cultures (Henrich et al., 2010; Rad et al., 2018). As such, further research is needed to examine whether and how the assumption of similarity among friends, as well as its impact on how people think and behave, differs across cultures and populations.

A growing body of research suggests that tracking the structure of the social world is vital to many aspects of everyday social thought and behavior (Brands, 2013; Brent, 2015; E. R. Smith & Collins, 2009; Weaverdyck & Parkinson, 2018). This requires accurately tracking others’ relationships (i.e., ties between third parties). The current research advances our understanding of these phenomena and could be integrated with research on other kinds of social knowledge, such as the inference of in-group/out-group boundaries (Lau et al.,2020, 2018), to gain a fuller picture of how we infer the structure of the social world in which we are embedded (Parkinson & Du, 2020). In addition, future research should explore the extent to which people assume that others will be connected with individuals who are similar to themselves with respect to additional behavioral tendencies and traits; such assumptions may be particularly strong with respect to cooperative tendencies, as in this study, given that the tendency for cooperators to sever ties with exploitative individuals may underlie broader cooperation (Izquierdo et al., 2014).

## Conclusion

All social interactions unfold within networks of social relationships. Here, we found evidence that people use third-party relationship knowledge when making trust decisions, even after having direct experience on which to draw. In addition, we found that expectations of behavioral similarity among friends shape mental representations of social networks. Taken together, these results suggest that similarity among friends serves as a social prior, causing knowledge of social networks and of others’ behavioral tendencies to reciprocally interact to shape social thought and behavior. This reciprocal relationship between beliefs about others’ behavioral tendencies and their relationships with one another suggests that the social decisions that people make are fundamentally intertwined with the networks of social relationships that they inhabit.

## Supplemental Material

sj-pdf-1-psp-10.1177_01461672221140269 – Supplemental material for Similarity Among Friends Serves as a Social Prior: The Assumption That “Birds of a Feather Flock Together” Shapes Social Decisions and Relationship Beliefs Supplemental material, sj-pdf-1-psp-10.1177_01461672221140269 for Similarity Among Friends Serves as a Social Prior: The Assumption That “Birds of a Feather Flock Together” Shapes Social Decisions and Relationship Beliefs by Miriam E. Schwyck, Meng Du, Yuchen Li, Luke J. Chang and Carolyn Parkinson in Personality and Social Psychology Bulletin

## Notes

1. In this experiment, we refer to “friend” as one of the partner’s “Top 3 Friends” on the gaming site where members could interact with one another in a variety of ways. Participants were told that members of the community regularly answered questions about how much they favored one another and how much they would like to engage with one another in the future; an algorithm allegedly computed each member’s “Top 3 Friends” based on who that member consistently favored and was favored by.
