# Supplementary material for: Similarity Among Friends Serves as a Social Prior: The Assumption That “Birds of a Feather Flock Together” Shapes Social Decisions and Relationship Beliefs
Source: Pers Soc Psychol Bull. 2023 Feb 2;50(6):823–40. doi: 10.1177/01461672221140269 (PMC11080385; doi:10.1177/01461672221140269)
Supplement: sj-pdf-1-psp-10.1177_01461672221140269 – Supplemental material for Similarity Among Friends Serves as a Social Prior: The Assumption That “Birds of a Feather Flock Together” Shapes Social Decisions and Relationship Beliefs [file sj-pdf-1-psp-10.1177_01461672221140269.pdf]

SUPPLEMENTAL MATERIAL TO ACCOMPANY

Similarity among friends serves as a social prior: The assumption that "birds of a feather flock together" shapes social decisions and relationship beliefs

This document has 6 sections:

S1. Learning partner trustworthiness in Block 1 trust games

Table S1. Linear mixed model on Block 1 offers over time

Figure S1. Participants successfully learned partner trustworthiness in Block 1

S2. Are people more influenced by knowledge of their partners' friends when their partners themselves are less predictable?

Table S2. Linear mixed model on coefficients of variation for trust game offers and partner preference ratings across levels of friendship knowledge in Block 2

Figure S2. People are influenced more by friend knowledge when their partners are unpredictable

S3. Examining the effects of shared demographic characteristics between participants and their partners

S4. Examining if people tended to forget ties among dissimilar partners

S5. Examining whether similarity-attraction was present

S6. Experimental instructions

S1. Learning partner trustworthiness in Block 1 trust games

Block 1 trust games were used for the sole purpose of manipulating participants' beliefs about the trustworthiness of future partners' friends. To verify that this manipulation was effective, we conducted a linear mixed effects model with offer amount as the dependent variable, and partner trustworthiness, both linear and quadratic transformations of time (Trial), and interactions between time and partner trustworthiness, as fixed effect predictors. Random intercepts and random by-trial linear slopes were included for each participant for each level of trustworthiness (Table S1). The main effect of partner trustworthiness showed that participants invested less in untrustworthy partners ($M = 0.95$, $SE = 0.13$) than in trustworthy partners ($M = 3.56$, $SE = 0.15$), $\Delta = 2.61$, 95% CI [2.33, 2.89], $t(3115) = 18.17$, $p < .001$, $d = 0.65$. Additionally, the interaction effects of partner trustworthiness with both the linear and quadratic transformations of time were significant, suggesting that participants successfully learned, over the course of the block, which Block 1 partners were trustworthy and which were not (Figure S1). Thus, we were able to assess the impact of this knowledge on their behavior towards new partners during Block 2.

Table S1

Linear Mixed Model on Block 1 Offers Over Time

Effect	β	95% CI	*F*	*DF1*	*DF2*	*p*	
Partner Trustworthiness	1.30	[1.16, 1.44]	330.07	1	3115	<.001	***
Trial	0.00	[-0.03, 0.02]	0.01	1	3115	.926	
Trial^2	0.01	[0.00, 0.01]	9.42	1	3115	.002	**
Partner Trustworthiness : Trial	0.14	[0.12, 0.16]	153.43	1	3115	<.001	***
Partner Trustworthiness : Trial^2	-0.03	[-0.03, -0.02]	150.80	1	3115	<.001	***

+ $.050 \leq p < .100$
* $.010 \leq p < .050$
** $.001 \leq p < .010$
*** $.000 \leq p < .001$

Note. Results from a linear mixed model on offers made in Block 1. The model included partner trustworthiness and both linear and quadratic transformations of time (centered Trial) as fixed effects, and random by-participant intercepts and by-participant linear slopes over time at each level of partner trustworthiness.

Figure S1

Participants Successfully Learned Partner Trustworthiness in Block 1

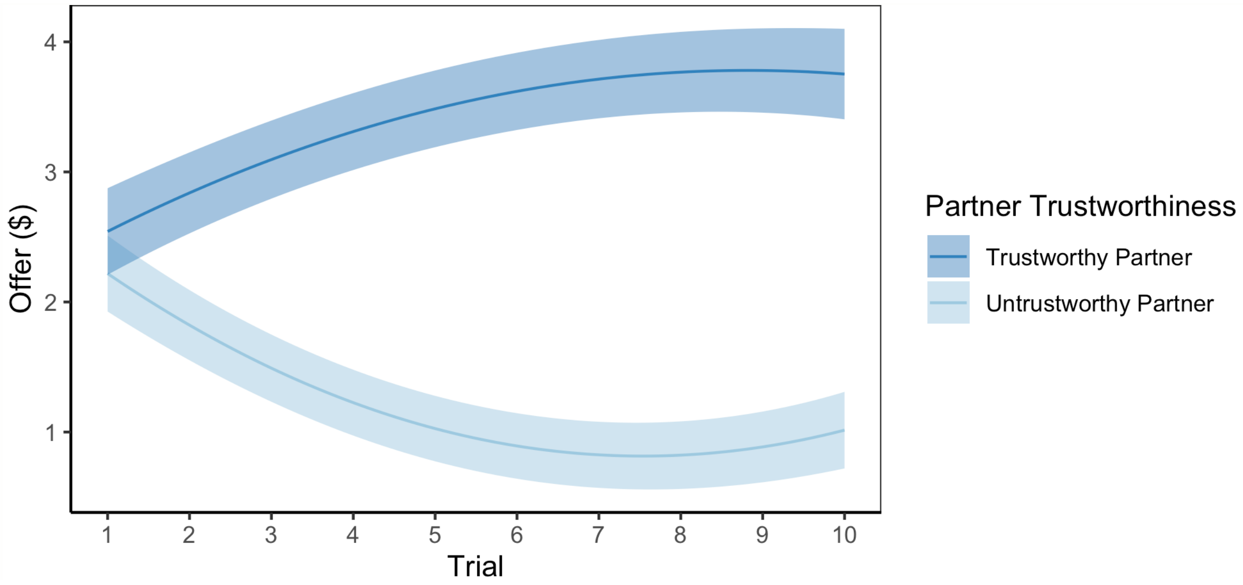

Note. Participants offered more money to partners who behaved in a trustworthy manner than to those who behaved in an untrustworthy manner, suggesting that they successfully learned which partners were trustworthy and which were not in Block 1. Error bars show 95% CI.

S2. Are people more influenced by knowledge of their partners' friends when their partners themselves are less predictable?

In our main analyses, we tested how the consistency of partners' behavior affected the use of friendship knowledge in trust game offers and partner ratings by including it in the full linear mixed effects models. The results of such analyses indicated, for example, that people were impacted by knowledge of their current partners' friends when interacting with *inconsistently* untrustworthy partners, but not when interacting with *consistently* untrustworthy partners, as described in the main text. That said, these analyses are limited to testing directed effects of partner variance and therefore would not detect an effect if participants varied in *how* they use friendship knowledge (see below for further discussion). Thus, we also conducted an exploratory analysis examining if people are generally more impacted by friendship knowledge when their partner is relatively unpredictable (i.e., when they have behaved inconsistently) than when their partner is more predictable (i.e., when their return rates have been very consistent). To examine the variability of offers made to Block 2 partners as a function of friend knowledge, we calculated the coefficient of variation (i.e., the ratio of the standard deviation to the mean) across mean offers to the three partners (one at each level of friend knowledge) for each participant within each unique combination of partner trustworthiness and variance. This yielded an estimate of how much each participant's offers varied as a function of friend knowledge within each unique combination of the remaining two conditions, which pertain to their partners' own behavior (partner trustworthiness and variance). We ran a linear mixed effect model on these data, with partner trustworthiness and variance as fixed effects, and random by-participant intercepts (Table S2). This analysis revealed a significant main effect of partner variance, such that participants varied their offers based on friend knowledge more when their partner was inconsistent ($M = 0.39$, $SE =$

0.03) than when their partner behaved consistently (*M* = 0.33, *SE* = 0.03; Table S2). Subsequent analyses of the estimated marginal means for each combination of condition levels revealed that this was true only in the trustworthy condition, Δ = 0.08, 95% CI [0.01, 0.15], *t*(237) = 2.14, *p* = .033, *d* = 0.28. A non-significant difference in the same direction was found when analyzing variability in the partner preference ratings in the same way (Table S2, Figure S2). Thus, knowledge of how a partner's friend had behaved influenced participants' behavior more when that partner was inconsistently, rather than consistently, trustworthy.

Table S2

Linear Mixed Model on Coefficients of Variation for Trust Game Offers and Partner Preference Ratings Across Levels of Friendship Knowledge in Block 2

Effect	β	95% CI	*F*	*DF1*	*DF2*	*p*	
Trust Game Offers							
Partner Trustworthiness	-0.01	[-0.04, 0.01]	0.86	1	237	.355	
Partner Variance	-0.03	[-0.05, -0.01]	5.75	1	237	.017	*
Partner Trustworthiness : Partner Variance	-0.01	[-0.03, 0.02]	0.40	1	237	.529	
Partner Preference Ratings							
Partner Trustworthiness	0.00	[-0.08, 0.08]	0.00	1	34	.998	
Partner Variance	-0.02	[-0.10, 0.06]	0.23	1	34	.632	
Partner Trustworthiness : Partner Variance	-0.01	[-0.09, 0.06]	0.15	1	34	.700	

+ .050 ≤ *p* < .100
* .010 ≤ *p* < .050
** .001 ≤ *p* < .010
*** .000 ≤ *p* < .001

Note. The coefficient of variation (i.e., the ratio of the standard deviation to the mean) across mean offers to (i.e., Trust Game Offers) and ratings of (i.e., Partner Preference Ratings) the three partners within each unique combination of partner trustworthiness and variance was calculated per participant, which provides an estimate of how much each participant's offers varied as a function of friend knowledge. One linear mixed model was run per task, and included partner trustworthiness and partner variance as fixed effects, along with random by-participant intercepts. Follow-up *t*-tests show that the friend knowledge influenced participants' offers more (i.e., participants' offers had a higher coefficient of variation) when playing with inconsistently compared to consistently trustworthy partners.

Figure S2

People Are Influenced More by Friend Knowledge When Their Partners Are Unpredictable

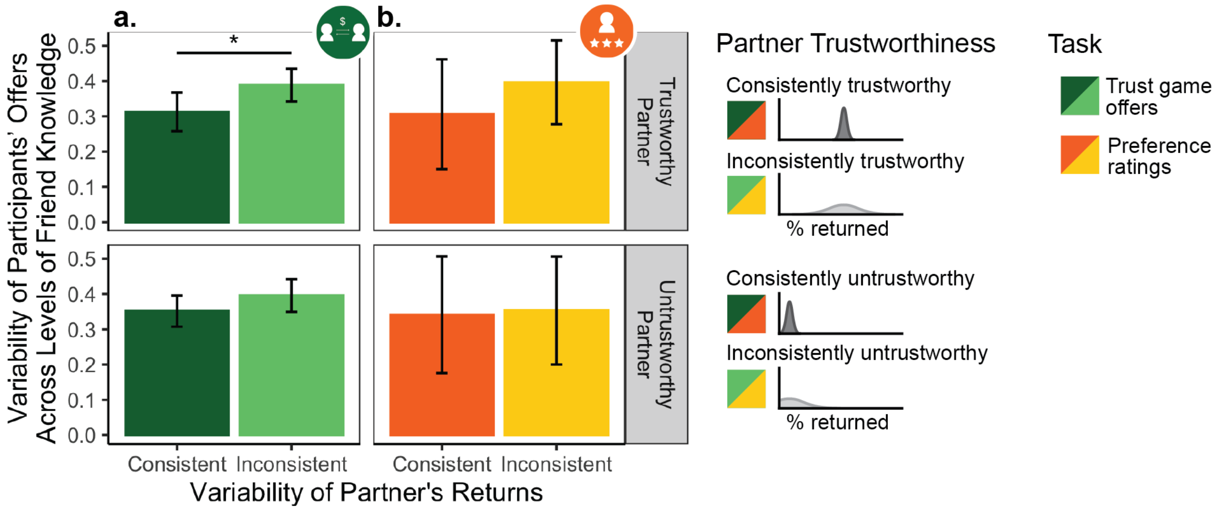

Note. The coefficient of variation (i.e., the ratio of the standard deviation to the mean) across **(a)** mean offers to and **(b)** preference ratings of the three Block 2 partners within each unique combination of partner trustworthiness and variance was calculated per participant. This provides an estimate of how much each participant's offers varied as a function of friend knowledge within each of the four cells and is plotted on the *y*-axis. As shown in **(a)**, friend knowledge influenced participants' offers more (i.e., higher coefficient of variation) when playing with inconsistent (compared to consistent) partners, $F(1, 237) = 5.75$, $p = .017$. This effect was driven by trustworthy partners, such that when participants played with trustworthy partners, their offers varied more as a function of their knowledge of a partner's friends if the partner behaved inconsistently (i.e., high-variability), rather than consistently (i.e., low-variability), $\Delta = 0.08$, 95% CI [0.01, 0.15], $t(237) = 2.14$, $p = .033$, $d = 0.28$. A similar, but non-significant, pattern of results is seen in partner preference ratings, as shown in **(b)**. Asterisks indicate significant differences between friend knowledge conditions (*$p < .050$), and error bars show 95% CI.

These results (which reflect how much, in general, participants' offers were influenced by friend knowledge for each kind of partner they played with, irrespective of how this influence unfolded within each participant) differ somewhat from the effects of partner variance noted in our main analyses. It should be noted that the analyses of participants' coefficients of variation described above are sensitive to the extent to which friend knowledge shaped behavior, but not the direction of such effects, whereas the effects of partner variance in the analyses described in the main text could only detect the influence of friend knowledge if it unfolded in a consistent direction across participants. As such, one possibility is that participants' behavior varied more as a function

of friend knowledge when their partners were inconsistently trustworthy (i.e., mean return rate = 50%; variance = 0.12) than when their partners were consistently trustworthy (i.e., mean return rate = 50%, variance = 0.01; Figure S2), but the precise nature of this relationship varied somewhat across participants. For example, when interacting with inconsistent but generally trustworthy partners, some participants may have been strongly influenced by expectations of similarity among friends that persisted across interactions, expecting people with trustworthy friends to be trustworthy themselves, even when their own behavior was variable. Contrastingly, other participants may have had particularly aversive responses to sometimes receiving relatively low offers from these same partners (as return rates for high-variance trustworthy partners dipped below 33% on some trials), especially when they had a strong prior expectation of fair behavior (i.e., especially when a trustworthy partner who was known to have trustworthy friends offered less than expected on a given trial). We also note that it may have been relatively difficult for some or all participants to learn that the inconsistently trustworthy partners were in fact trustworthy, given that although their return rates were drawn from a distribution centered on 50%, this distribution was relatively wide and included offers that were considerably lower than 50%. Thus, 10 trials with each interaction partner in Block 2 may not have been sufficient for all participants to learn that high-variance, trustworthy partners were in fact trustworthy (consistent with Figure 3b-c). It is also possible that for many people, the concepts of trustworthiness and behavioral consistency are fundamentally intertwined, and thus, two partners with equivalent return rates (e.g., 50%) but different levels of variability across trials may not always be perceived as equivalently trustworthy, even if the central tendency of their behavior is equivalent across trials. Thus, further research is needed to better understand the relationship between behavioral variability, social network knowledge, and expectations about how other people will behave.

S3. Examining the effects of shared demographic characteristics between participants and their partners

All stimulus images were randomly assigned to partner profiles to negate any potential effects of demographics on trust behavior. For completeness's sake, and to explore any possible influence these incidental social categories may have had on participants' behavior, we reran all of the linear mixed models examining trust game offers with covariates accounting for shared race and gender between the participant and each partner. The stimuli were categorized as Asian, Black, Latinx, or White according to the Chicago Face Database. Participants were categorized into the same four categories ($N_A = 39$, $N_B = 3$, $N_L = 14$, $N_W = 16$), along with a fifth category ($N = 8$) for participants who did not self-identify as one of these. For each participant, a binary variable was created to code for whether or not the participant and the partner were of the same race. Similarly, the stimuli were categorized as male or female in the Chicago Face Database, and a second binary variable was created to code for whether or not the participant and the partner identified as the same gender.

Having the same race as one's partner did not significantly impact offers made in block 1 ($M_{same} = 2.23$, $SE = 0.13$; $M_{different} = 2.25$, $SE = 0.12$), $\Delta = 0.02$, 95% CI [-0.13, 0.08], $t(3113) = 0.47$, $p = .638$, $d = 0.02$, or block 2, for either initial offers ($M_{same} = 2.64$, $SE = 0.16$; $M_{different} = 2.53$, $SE = 0.16$), $\Delta = 0.11$, 95% CI [-0.01, 0.23], $t(876) = 1.75$, $p = .080$, $d = 0.12$, or offers over time ($M_{same} = 1.96$, $SE = 0.13$; $M_{different} = 1.96$, $SE = 0.13$), $\Delta = 0.01$, 95% CI [-0.05, 0.07], $t(9483) = 0.28$, $p = .785$, $d = 0.01$. Similarly, having the same gender as a partner did not significantly predict offers made in block 1 ($M_{same} = 2.22$, $SE = 0.12$; $M_{different} = 2.26$, $SE = 0.12$), $\Delta = 0.03$, 95% CI [-0.05, 0.12], $t(3113) = 0.74$, $p = .460$, $d = 0.03$, or initial offers in block 2 ($M_{same} = 2.58$, $SE = 0.16$; $M_{different} = 2.59$, $SE = 0.16$), $\Delta = 0.01$, 95% CI [-0.09, 0.11], $t(876) = 0.19$, $p = .847$, $d = 0.01$;

however, there was a significant effect on offers over time, such that participants offered more to players of different genders (M_{same} = 1.92, *SE* = 0.13; $M_{different}$ = 2.00, *SE* = 0.13), Δ = 0.08, 95% CI [0.03, 0.13], *t*(9483) = 2.97, *p* = .003, *d* = 0.06. Furthermore, the patterns of results discussed in the main text of the manuscript remained the same with and without the added covariates.

It is of particular interest that neither gender nor race impacted offers made to new partners in block 2, but friendship knowledge did. Importantly, the current study was not designed to test effects of demographic knowledge on trust behavior, and thus we interpret these results with extreme caution. These findings do suggest, however, that when encountering someone for the first time, knowledge of that person's relationships with others may play a particularly important role in impression formation. As such, future research examining effects of perceived social categories on cognition and behavior may benefit from considering the effects of social network information.

S4. Examining if people tended to forget ties among dissimilar partners

In our main analyses, we examined if the assumption of a link between behavioral similarity and friendship colored mental representations of social relationships by examining false alarms, consistent with previous work that has examined the impact of schemas on memory of non-social contents (Brewer & Treyens, 1981; De Brigard et al., 2017; Lampinen et al., 2001). For completeness, we also tested if assumptions of a link between behavioral similarity and friendship shaped the extent to which participants forgot friendships that did exist (i.e., false negative rate). We ran a linear mixed model with false negative rate (arcsine transformed) as the dependent variable, dyad trustworthy similarity, dyad variance similarity, and their interaction as fixed effects, and random by-participant intercepts. We did not find any significant main or interaction effects in this analysis, $\beta_{\text{trustworthiness similarity}}$ = 0.04, 95% CI [-0.01, 0.10], $F(1, 237) = 2.19$, $p = .140$; $\beta_{\text{variance similarity}}$ = 0.02, 95% CI [-0.04, 0.07], $F(1, 237) = 0.37$, $p = .542$; $\beta_{\text{interaction}}$ = -0.02, 95% CI [-0.07, 0.04], $F(1, 237) = 0.37$, $p = .542$. That said, consistent with the pattern of results discussed in the main text, we found that the marginal mean false negative rates tended to be slightly higher for dyads composed of people who were dissimilar in their trustworthiness compared to dyads composed of people who were similar to each other. This was true for both pairs of people who behaved in a consistent manner ($M_{\text{dissimilar}} = 0.85$, $M_{\text{similar}} = 0.81$), and those composed of one consistently-behaving and one inconsistently-behaving partner ($M_{\text{dissimilar}} = 0.85$, $M_{\text{similar}} = 0.74$). (Note, since all Block 1 partners were consistent, there were no known friendships that consisted of two inconsistent partners.) That is, people tended to be slightly more likely to forget that two people were friends if they behaved dissimilarly. Importantly, these effects did not reach significance and should thus be interpreted with caution; thus, further research is needed to confirm and clarify this effect.

S5. Examining whether similarity-attraction was present

The primary question of this manuscript asks if people *assume* that friends will be exceptionally similar to one another when interacting with others. A separate question is: Does similarity-attraction (i.e., preference for similarly-behaving others) exist in the context of our experiment? We are unable to directly test this question with the current data because participants always played the role of Player 1 in the trust game, but they only observed others play the role of Player 2. Therefore, we have data on how much participants tended to place *their* trust in others (i.e., how *wary/trusting* they were of others), but participants did not learn how *wary/trusting* their partners were of others. Similarly, while participants learned how *trustworthy* their partners were, we do not have data on how trustworthy participants were themselves. One's own trustworthiness and how wary one is of trusting others are distinct qualities: For example, someone could be both cautious when it comes to placing their trust in new people and still be unlikely to betray others' trust themselves (i.e., wary but trustworthy); someone could also be both cautious when it comes to placing their own trust in new people and likely to betray others trust (i.e., wary and untrustworthy). We suggest that future work augment the current paradigm by (1) also having participants observe their partners be Player 1 in the trust game, and/or (2) by measuring participants' behavior when they assume the role of Player 2 in the trust game. This would provide data on (1) both participants' own wariness and others' apparent wariness (i.e., how readily they trust people) and/or (2) both participants' own trustworthiness and the apparent trustworthiness of others. Such data would allow future work to build on the current study, which tests if people assume that others will be similar to their friends, by testing the separate but complementary questions of (1) if this assumption reflects reality and (2) if the degree to which

people expect others to behave similarly to their friends is related to the extent to which they themselves prefer to interact with and befriend similar others.

All of that being said, we conducted exploratory analyses testing if the extent to which people were wary of trusting others was related to the extent to which they preferred partners who are trustworthy (i.e., examine associations between participants' behavioral tendencies regarding how *wary/trusting* they were of others, and their preference ratings of others who were differentially *trustworthy*). We ran a linear mixed model with participant wariness (calculated as their average offer to all partners, such that higher average offers reflect less wariness), partner trustworthiness, and their interaction predicting partner preference ratings (*z*-scored within each participant) with random by-participant slopes. If participants who are less wary (i.e., more trusting) prefer trustworthy partners *and* more wary participants (i.e., less trusting) prefer untrustworthy partners, then we should see a significant interaction effect. However, only partner trustworthiness significantly predicts partner preferences, $\beta = 0.63$, 95% CI [0.55, 0.72], $F(1, 1198) = 208.90$, $p < .001$, such that trustworthy partners are preferred above untrustworthy partners by everyone. As noted above, people who are trusting/wary of others are not necessarily trustworthy/untrustworthy themselves, and vice versa. As such, we cannot speak confidently as to whether or not participants preferred those who were more similar to themselves.

Although it is not possible to draw conclusions about the existence of similarity-attraction based on these results, given the limitations of the data noted above, this data analytic approach could be adopted in future work to test for similarity-attraction (e.g., to test if the trustworthiness of participants is related to their preference for trustworthy partners and/or if participants' wariness of placing their trust in others is related to their preference for others who are similarly wary of placing their trust in others).

S6. Experimental instructions

Excerpts from the onscreen instructions provided to participants are included below. The full paradigm is available online at https://anonymous.4open.science/r/c4145239-6a86-47b9-a681-a063184ec182.

"Welcome to our social gaming website! You are participating in a study that examines how various factors, like the amount of reward that's at stake, impact players' enjoyment of online games. You'll be playing a series of simple games with other people on this website. We're currently testing out this website with collaborators at other colleges in the U.S. The players you'll be partnered with today are students at other colleges who regularly play a variety of games with one another for fun and the chance to earn prizes (e.g., money). Users of the site regularly have the chance to rate each other in terms of how much they prefer playing with one another. Players who consistently choose one another as their favorite partners to play with on the site are called one another's "Top Friends". When members of this website play with one another, their 3 Top Friends are displayed below their own profile photo…. Since this is your first time using this website, you won't yet have any Top Friends displayed alongside your profile picture. Due to most users' privacy settings, new users like you can only see those friends' faces if you've interacted with them before on the site.

….

Please wait while we assign games for you...

In Block X, you are assigned to play the Investment Game with other players. In each round of the Investment Game, you'll be partnered with one person.

Please wait while we assign your role...

You've been assigned the role of first player.

….

You have now completed Block 2! We really want to hear about your enjoyment of the games you played today. To ensure that your session today doesn't run overtime, we're now going to move on to the post-game feedback session rather than having you complete Block 3."
